# A change in the zinc ion concentration reflects the maturation of insulin-producing cells generated from adipose-derived mesenchymal stem cells

**DOI:** 10.1038/s41598-019-55172-0

**Published:** 2019-12-10

**Authors:** Shogo Ohta, Tetsuya Ikemoto, Yuma Wada, Yu Saito, Shinichiro Yamada, Satoru Imura, Yuji Morine, Mitsuo Shimada

**Affiliations:** Department of Digestive and Transplant Surgery, Tokushima University, 3-18-15 Kuramoto, Tokushima, 770-8503 Japan

**Keywords:** Differentiation, Mesenchymal stem cells, Stem-cell differentiation, Type 1 diabetes

## Abstract

The generation of insulin-producing cells (IPCs) from pluripotent stem cells could be a breakthrough treatment for type 1 diabetes. However, development of new techniques is needed to exclude immature cells for clinical application. Dithizone staining is used to evaluate IPCs by detecting zinc. We hypothesised that zinc ion (Zn^2+^) dynamics reflect the IPC maturation status. Human adipose-derived stem cells were differentiated into IPCs by our two-step protocol using two-dimensional (2D) or 3D culture. The stimulation indexes of 2D -and 3D-cultured IPCs on day 21 were 1.21 and 3.64 (*P* < 0.05), respectively. The 3D-cultured IPCs were stained with dithizone during culture, and its intensity calculated by ImageJ reached the peak on day 17 (*P* < 0.05). Blood glucose levels of streptozotocin-induced diabetic nude mice were normalised (4/4,100%) after transplantation of 96 3D-cultured IPCs. Zn^2+^ concentration changes in the medium of 3D cultures had a negative value in the early period and a large positive value in the latter period. This study suggests that Zn^2+^ dynamics based on our observations and staining of zinc transporters have critical roles in the differentiation of IPCs, and that their measurement might be useful to evaluate IPC maturation as a non-destructive method.

## Introduction

The generation of insulin producing cells (IPCs) derived from pluripotent stem cells (PSCs) has the possibility to resolve the issue of donor shortages for islet transplantation in some countries^1^. Many studies have investigated the induction of IPCs from PSCs, such as embryonic stem cells, induced pluripotent stem cells, and other organ-derived cells^2–11^. In particular, adipose-derived stem cells (ADSCs) can be procured less invasively and without ethical problems compared with some types of PSCs^12^. An advantage of ADSCs is that up to 300-fold more stem cells can be obtained from the same quantity of bone marrow aspiration^13^. Moreover, they might be easy to apply clinically because such cell transplantation is considered auto-transplantation that does not require immunosuppression. Thus, we focused on ADSCs as a new cell source for IPCs^12,14,15^. We have previously reported a new two-step differentiation protocol^16^ and its modification to a more effective xeno-free and three-dimensional culture protocol^17^ to generate functional IPCs from ADSCs. For clinical application, it is important to exclude immature IPCs. Moreover, IPC maturation has been induced *in vivo* at a few months after transplantation^3,18^. Thus, the best timing for IPC transplantation is still unclear.

The role of the zinc ion (Zn^2+^) in pancreatic β-cells is important for basic cell structures and enzymes. Mammalian pancreatic β-cells contain higher levels of intracellular Zn^2+^ than other organs^19^. In β-cells, insulin binds to Zn^2+^ and is converted into a hexamer within secretory granules^20^. Then, Zn^2+^ is released with insulin and absorbed by β-cells again. Therefore, mature β-cells both take up and Zn^2+^ secrete^21,22^. Dithizone staining is used to detect islets by the presence of high density Zn^2+^, which can be applied to evaluate IPCs^8,16,20^. However, cells are destroyed by the toxicity of dithizone in this procedure, and it is dangerous to use dithizone clinically because of its carcinogenicity^8,20^.

Zn^2+^ is an important metal ion related to a variety of metabolic functions. For example, DNA and RNA polymerases, which are necessary for cell proliferation, gene expression, and matrix metalloproteases, which operate in cell migration and invasion, are Zn^2+^-dependent enzymes^23–25^.

It has been hypothesised that Zn^2+^ might be absorbed for cell activity during differentiation and maturation, and secreted with insulin upon maturation. Therefore, Zn^2+^ dynamics in differentiated cells are considered as the novel marker for the maturation of generated IPCs.

Here, we determined whether the Zn^2+^ concentration in culture medium is an easy and useful marker of IPC differentiation and maturation, and demonstrate the mechanism of Zn^2+^ dynamics of IPCs.

## Results

### IPCs in 3D culture form cell clusters more easily than in conventional 2D culture

**Figure 1:**
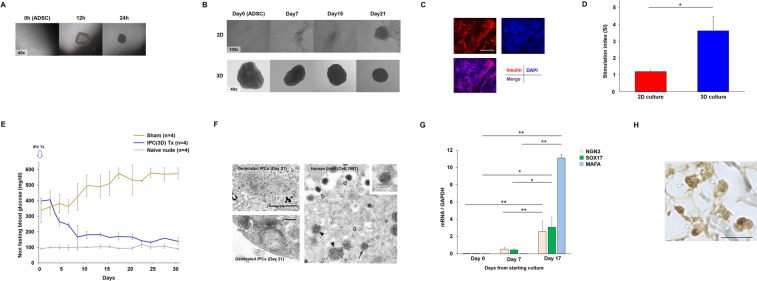
(**A**) ADSCs formed cell clusters within 24 hours after adding RCPγ pieces. Original magnification x40. (**B**) Cell cluster-like formation required more time in conventional 2D culture (upper row, original magnification x100) compared with 3D culture (lower row, original magnification x40). **(C)** Stimulation index of 3D-cultured IPCs was significantly higher than that of 2D-cultured IPCs on day 21 (*P = 0.004, Welch’s test). **(D)** Mature IPCs expressed insulin strongly according to immunofluorescence staining. Day 21, 3D-cultured IPCs. **(E)** Blood glucose levels of streptozotocin-induced diabetic nude mice changed to normoglycaemia levels after IPC transplantation (blue line, n = 4), whereas the sham mouse group (ochre line, n = 4) did not reach a normal range. Grey line shows blood glucose levels of naïve nude mice (n = 4). **(F)** Electron microscopy revealed dense cystic microstructures (left, upper row. Scale bar, 5 μm) and secretory granule-like structures (left, lower row. Scale bar, 0.2 μm) in the cytoplasm of 3D-cultured IPCs on day 21, resembling secretory granules observed in human naïve β-cells^25^ (right). White arrowhead: mature granules; black arrowhead: clathrin-coated secretory vesicles; arrow: condensing secretory material in a clathrin-coated Golgi cisterna. **(G)** RT-PCR analyses of SOX17, NGN3, and MAFA expression on days 0, 7. and 17 (*P < 0.01, **P < 0.001, Bonferroni’s test). **(H)** Immunohistochemistry of 3D-cultured IPCs on day 21 showed strong insulin expression (arrowhead). R, RCPγ piece. Scale bar, 25 μm.

During two-step differentiation, there were some morphological differences between the conventional 2D culture and 3D culture due to the culture duration. In detail, in the 3D culture, ADSCs and RCP pieces gathered and underwent sphere-like formation within 24 hours of culture (Fig. 1A). Subsequently, IPCs generated in 3D culture exhibited sphere-like formations until 21 days, whereas cell clusters had formed at around day 21 in conventional 2D culture (Fig. 1B). Immunofluorescence staining detected human insulin in the cytoplasm of 3D-cultured IPCs on day 21 (Fig. 1C). These culture method-related differences revealed that the stimulation index (SI) was significantly higher in 3D-cultured IPCs compared with conventional 2D culture on day 21 (3.64 ± 0.86 vs. 1.21 ± 0.11, *P* < 0.05, Welch’s test, Fig. 1D). Measured insulin concentrations including under the non-stimulated (basal) condition of each culture system are shown in Table 1.

**Table 1:** Insulin concentrations (pM) in culture medium supernatant.

	Base line (pM)	Low glucose (pM)	High glucose (pM)	SI
Conventional culture (2D, n = 3)	18.1 ± 2.0	7.5 ± 2.6	8.9 ± 2.7	1.2 ± 0.1
3D culture (n = 3)	16.4 ± 1.1	34.3 ± 22.5	129.9 ± 100.2	3.6 ± 0.9

Note: all values are presented as the mean ± S.D.

### 3D-cultured IPCs reduce blood glucose levels of DM mice after transplantation

As an *in vivo* functional assay, after transplantation of 96 3D-cultured IPCs into the mesentery of STZ-induced DM nude mice (n = 4), blood glucose levels gradually decreased below 200 mg/dl. Briefly, at 6 days after transplantation, the blood glucose levels of four mice decreased to under 200 mg/dl and were maintained below this level until 30 days after transplantation (4/4,100%), whereas the sham group (n = 4) could not convert their hyperglycaemic state to a normoglycaemia level (Fig. 1E).

### 3D-cultured IPCs contain secretory granules and secrete insulin

Electron microscopy showed insulin secretory granule-like structures and dense structures in 3D-cultured IPCs on day 21 as observed in human naïve β-cells as the control (Fig. 1F).

### mRNA expression of differentiation marker genes in 3D-cultured IPCs

Expression of SOX17 as an endoderm development marker, of NGN3 as an endocrine cell differentiation marker, and of MAFA as an index of mature pancreatic β-cells, on day 17 were significantly higher compared with days 0 (SOX17, *P* < 0.01; NGN3 and MAFA, *P* < 0.001, Bonferroni’s test, Fig. 1G) and day 7 (SOX17, *P* < 0.01; NGN3 and MAFA, *P* < 0.001, Bonferroni’s test, Fig. 1G).

### Immunohistochemistry of IPCs showed strong insulin expression

To clarify the expression of insulin, IPCs were investigated using immunohistochemistry. The cytoplasm of IPCs in day 21 3D cultures stained strongly (especially granules-like structures) for insulin (Fig. 1H).

### Dithizone stains IPCs gradually over time

**Figure 2:**
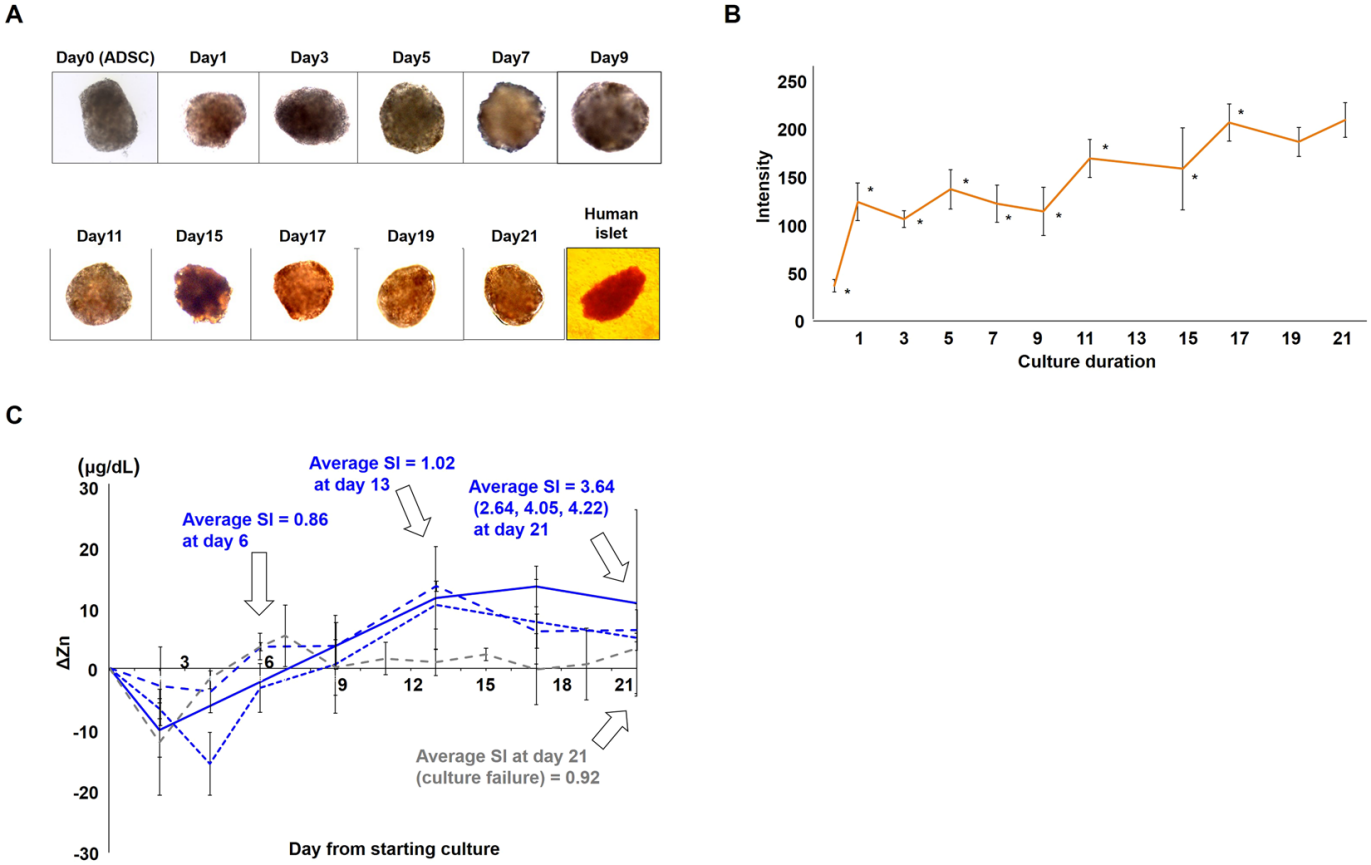
(**A**) Sphere-like formations containing ADSCs were gradually positively stained by dithizone every 2 days during culture. Representative cell formations (n = 8 each time point) are shown. Freshly isolated human islets were included as a positive control (lower right corner). **(B)** Dithizone-stained cells were analysed by Image J. The staining intensity increased gradually and reached the peak on day 17. The average of three independent experiments is shown. *P < 0.01, Bonferroni’s test. Error bars represent the standard deviation. **(C)** Pattern of Zn^2+^ concentration changes in culture supernatants under 3D culture conditions. ΔZn^2+^  = Zn^2+^ (supernatant) − Zn^2+^ (fresh culture medium). Three independent experiments are shown as blue lines. Average SI at day 6, day 13 and day 21 are shown. Error bars represent the standard deviation. Grey-dotted line shows when cell culture failed to differentiate and mature properly because of the culture conditions. Average SI at day 21 was showed. Error bars represent the standard deviation.

In the 3D culture, the overall appearance of sphere-like formations was gradually stained by dithizone (Fig. 2A, eight IPCs observed at each point). Representative images of four independent experiments are shown in Fig. 2A. Quantification of images of cultured cells by ImageJ^26^ showed that the staining intensity was gradually increased up to 180 and had statistically peaked on day 17 (*P* < 0.01, Bonferroni’s test) Based on three random points of eight IPCs, the average value is shown in Fig. 2B. After day 17, there were statistically no significant differences in the staining intensity of dithizone. As a reference, we evaluated the intensity in images of isolated human islets (Fig. 2A, right corner of the lower row), which was 244.

### Zn^2+^ concentrations in culture medium change from negative to positive during proper IPC differentiation

Zn^2+^ concentration changes in the culture supernatants [ΔZn^2+^: calculated as Zn^2+^ (supernatant) – Zn^2+^ (fresh culture medium)] of the 3D-cultured IPCs were measured and plotted. In the early period of days 3 and 6, ΔZn^2+^ had a negative value. However, ΔZn^2+^ because positive from day 6 to 9 and reached its peak at day 13–17 (Blue lines in Fig. 2C, three independent experiments, SIs of each experiment were 2.64, 4.05, and 4.22, and the average SI was 3.64 at day 21, 0.86 at day 6, and 1.02 at day 13). However, if we failed to differentiate and properly mature cells because of a lack of supplements, the increase of ΔZn^2+^ was low (grey-dotted line, Fig. 2C, average SI was 0.92 at day 21).

### Zinc transporter expression in the early phase of the IPC differentiation protocol

**Figure 3:**
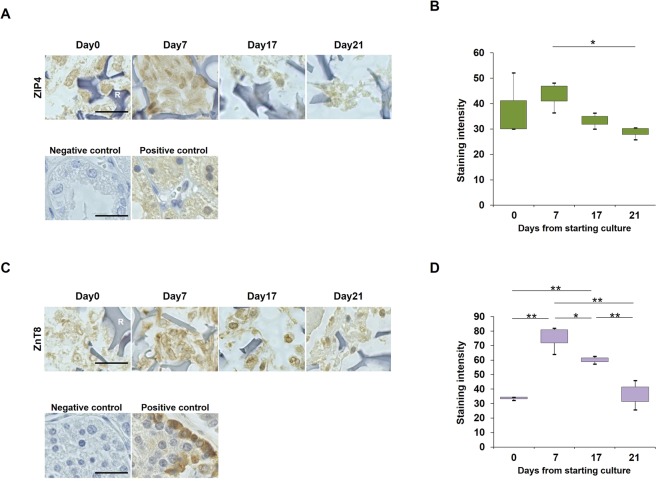
**(A)** Immunohistochemistry of ZIP4 at days 0, 7, 17, and 21. Representative results from three independent experiments are shown. **(B)** Staining intensities in images were measured by ImageJ. **(C)** Immunohistochemistry of ZnT8 at days 0, 7, 17, and 21. Representative results from three independent experiments are shown. **(D)** Staining intensities in images were measured by ImageJ. Three random fields were analysed. R: RCP petaloid μ-piece, *P < 0.05, **P < 0.01, Fisher’s least significant difference. Error bars represent the standard deviation.

ZIP4 expression was detected throughout the protocol, but reached its peak on day 7 and then decreased gradually (P < 0.05, vs day 21, Fisher’s least significant difference, Fig. 3A,B). However, ZnT8 expression was strongly detected from day 7 and then decreased significantly (P < 0.01, vs each point. P < 0.05, vs day 17, Fisher’s least significant difference, Fig. 3C,D).

### Positive change pattern of the Zn^2+^ concentration might be specific, but a negative change pattern might be general in cells differentiated from ADSCs

**Figure 4:**
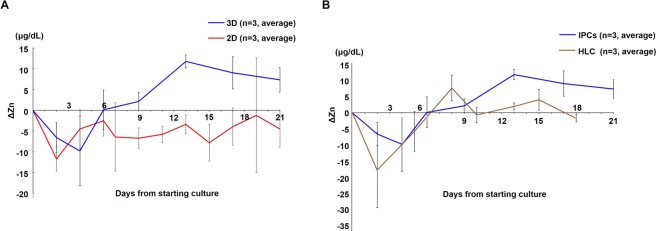
(**A)** Comparison of the pattern of Zn^2+^ concentration changes in the culture supernatant of IPCs (n = 8 at each point) under 3D culture and 2D culture conditions, **(B)** or hepatocyte-like cell (HLC, n = 8 at each point) differentiation. Three independent experiments were performed. Error bars represent the standard deviation.

ΔZn^2+^ of 3D-cultured IPCs reached its peak at day 13–17 as described above. However, ΔZn^2+^ in the conventional 2D culture had an obviously different pattern from that in the 3D culture, especially in the positive curve area (Fig. 4A). In the 3D culture, an increase of ΔZn^2+^ during day 7–21 was statistically correlated with the quantified intensity of dithizone staining (r = 0.798, Spearman’s rank correlation coefficient). To estimate the meaning of the negative curve area, the Zn^2+^ concentration changes were measured in the supernatant of hepatocyte-like cells (HLCs) cultured in differentiation culture medium. Interestingly, the Zn^2+^ concentration change of culture supernatant in in early stage of the HLC differentiation protocol was also negative, and the positive curve pattern was different from that of IPCs in 3D and 2D cultures (Fig. 4B).

## Discussion

Islet transplantation is a β-cell replacement therapy that has the potential to make type 1 diabetes mellitus (T1DM) patients free of insulin injections. However, islet transplantation is still not widespread because of some issues. One of them is the severe donor shortage in some countries such as Japan^1,27^. Moreover, repeat transplantation is often required to achieve an insulin-free status, which accelerates the donor shortage. To resolve this issue, it is desirable to establish more stable and safe cell sources. Therefore, insulin-producing cells (IPCs) derived from mesenchymal stem cells are expected to be an alternative and non-cadaveric therapeutic cell source.

For clinical application of IPCs, cells function should be evaluated by a non-destructive procedure before transplantation. In previous methods including dithizone staining, RT-PCR, immunofluorescence staining, and western blotting, large numbers of IPCs must be sacrificed. It is well known that Zn^2+^ plays a critical role in insulin release metabolism, and glucose stimulation induces Zn^2+^ release from islet cells^28^. Thus, we focused on the Zn^2+^ concentration because evaluation of the culture supernatant is a non-invasive method for cells. In this study, we found that the peak of dithizone staining intensity was day 17 and elevated differentiation marker expression. Interestingly, the staining intensity of generated IPCs approached that of human islets. However, as indicated by the SI, the generated IPCs had not completely achieved the same functions of human islets yet. Remarkably, ΔZn^2+^ did not reach statistical significance after day 17. Therefore, the maturation of IPCs might be adequate for transplantation earlier than day 21. Moreover, if we did not differentiate and mature IPCs properly because of the medium conditions, ΔZn^2+^ did not have a high enough positive value and a sufficient SI could not be achieved. These findings suggested that ΔZn^2+^ as an indicator of zinc ion dynamics in the culture medium reflected the IPC functional maturation status. Therefore, IPC transplantation on day 17 might produce interesting results.

However, the weekly increase in SI seemed to be different from that of zinc ion concentration in step 2. This observation suggested a discrepancy between the creation of insulin secretory granules and the acquisition of the ability to release insulin (ability to detect a change of glucose concentration), and this sensory ability might be obtained in the later phase of IPC differentiation.

**Figure 5:**
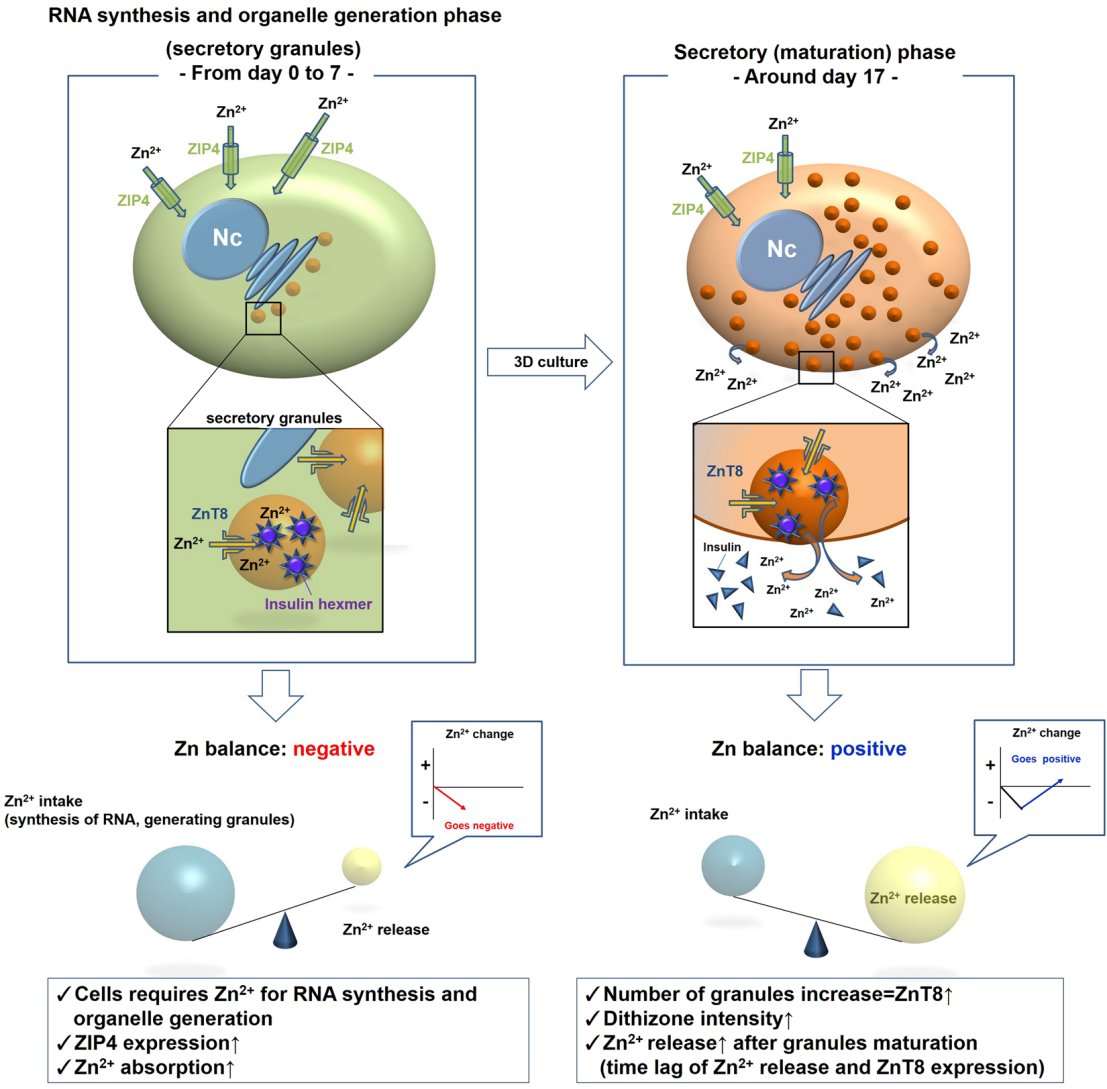
ADSC started to generate insulin secretory granules after induction to endoderm-derived cells and fate decision. To achieve these events, mass RNA synthesis is required. Thus, it was considered that ZIP4 was expressed and performed an essential role in cell development (“RNA synthesis and organelle generation phase”, left). Then, along with increases of secretory granules containing insulin hexamers with two Zn^2+^ each and expression of ZnT8, the intensity of dithizone staining was increased. After sufficient maturation, many secretory granules containing insulin and Zn^2+^ were gradually released from IPCs. Such release resulted in an increase of the Zn^2+^ concentration (“Secretory phase”, right).

Recently, Zn^2+^ transporters have been focused on for their roles in cells because Zn^2+^ is a functional component in many cellular proteins and enzymes, and plays a key role in cell growth and development^29–31^. Zn^2+^ transporters include the zinc transporter protein (ZnT)/solute carrier 30A (SLC30A) family and Zrt, Irt-like protein (ZIP)/SLC39A family. Many reports indicate that the ZnT family carries Zn^2+^ from the cytoplasm to organelles or extracellular regions, while the ZIP family carries Zn^2+^ retrodirectionally^21,30–32^. Among them, ZnT8 expression is detected mainly in insulin secretory granules^33–35^. Some T1DM cases are caused by an autoimmune disorder against ZnT8^36–41^. In a recent study, ZnT8 expression was correlated with insulin secretion in rat islet cell line INS-1E^42^. In addition, it has also been reported that ZIP4 expression is associated with intake of Zn^2+^ by islet β cells^43^ and their differentiation and maturation^44,45^. In previous reports, stem cells have absorbed Zn^2+^ in their nuclei during differentiation^46,47^. In HLCs derived from ADSCs, we showed that ΔZn^2+^ values were negative in the early period of differentiation. This result suggest that ADSCs require a large amount of Zn^2+^ in the early stage of differentiation, at least for differentiation into endodermal cells, which is probably used to generate organelles and synthesise RNA^47,48^. Moreover, Zn^2+^ absorption appears to occur in embryonic stem cells during endoderm transition^46,48^. Therefore, based on our results above and previous reports, we predicted the mechanism of Zn^2+^ changes during IPC differentiation (Fig. 5). Briefly, ADSCs start to generate insulin secretory granules after induction to endoderm and cell fate decision. To achieve these events, a large degree of RNA synthesis is required. Thus, ZIP4 performs an essential role for cell development^44,45^, which we named the “RNA synthesis and organelle generation phase” (Fig. 5 left). Then, along with increases of secretory granules, which contain insulin hexamers with two Zn^2+^ and ZnT8^34,35^, the intensity of dithizone staining increases. After sufficient maturation, many secretory granules containing insulin and Zn^2+^ are gradually released from IPCs, resulting in an increase of the Zn^2+^ concentration, which we named the “secretory phase”.

Regarding the limitations of this study, there are some issues to be investigated further. First, essentially, there are no fixed evaluation methods for IPC differentiation and maturation. Thus, it is still unclear whether these IPCs differentiate and mature sufficiently. Second, in our study, marker gene expression was evaluated by RT-PCR only. Therefore, marker gene expressions should be confirmed by western blotting and immunostaining. Similarly, zinc transporter protein expression was measured by immunohistochemistry only. By performing further experiments, the results of this study may confirm our hypothesis (Fig. 5). Third, during the differentiation of stem cells, further analyses are required to understand zinc ion dynamics. According to our results from HLCs as another cell type, at least ADSCs require a large amount of Zn^2+^ to convert into cells of other germ layers such as endoderm and generate organelles. Zinc is essential and required for cell differentiation^44,45^. Thus, zinc ion dynamics might reflect cell fate decisions. Therefore, evaluating Zn^2+^ dynamics in the differentiation of other germ layer cells might support our hypothesis (Fig. 5). Fourth, the precise microstructures of IPCs are still unknown. We observed some undefined “empty granules” in 3D-generated IPCs by electron microscopy; however, further investigations are required to determine whether these structures are special IPCs structures or generated as a result of cell preparation or storage, or cell damage.

In conclusion, Zn^2+^ concentration changes in the culture supernatant might be a marker of IPC maturation. This new insight might be useful in validating the function of transplantable IPCs, excluding inadequate IPCs for transplantation, or monitoring production.

## Materials and Methods

### ADSC preparation

STEMPRO™ Human Adipose-Derived Stem Cells (Invitrogen, Grand Island, NY) were used in this study. ADSCs were cultured in ADSC basal medium consisting of MesenPROTM RS (Gibco, Carlsbad, CA) with GlutaMAXTM-I (Gibco).

### IPC differentiation protocol

IPCs were generated as reported previously^16,17^. Briefly, after sufficient passaging, ADSCs were mixed with recombinant peptide micro-pieces (RCP μ-pieces; FUJIFILM, Tokyo, Japan) and transferred to a Nunchlon Sphera 96U Bottom Plate (Thermo Scientific, Waltham, MA) for 3D culture. For conventional 2D culture, ADSCs without RCP μ-pieces were seeded into FALCON™ 6 Well Flat Bottom Tissue Culture Plate with a Low Evaporation Lid (Corning Co., Corning, NY). The protocol for IPC induction has been reported previously^18^. However, the contents of the differentiation cocktail were changed to recombinant human activin-A, hepatocyte growth factor, and albumin. ADSCs were cultured using the step-1 (from day 0 to 7) medium consisting of Dulbecco’s modified Eagle’s medium/F12 (Gibco) with 1% recombinant human albumin (Wako, Osaka, Japan), 10 nM exendin-4 (Sigma-Aldrich, St. Louis, MO), 1% N2 supplements (Gibco), 1% B27 supplement (Gibco), and 50 ng/mL recombinant human activin-A (PeproTech, Rocky Hill, NJ). Step-2 (from day 8 to 21) medium was the same as step-1 medium with addition of 50 ng/mL recombinant human hepatocyte growth factor (PeproTech), valproic acid (Wako) and 10 mM nicotinamide (Sigma-Aldrich). The culture medium was changed and the supernatant was collected every 2 days.

### Hepatocyte-like cell differentiation protocol

Hepatocyte-like cells (HLCs) were differentiated as reported previously^49^. Briefly, ADSCs were cultured using a 20-day differentiation protocol under 2D culture conditions. The differentiation protocol employed 2 μM Chir99021 (MedChemExpress, Monmouth Junction, NJ, USA), 1% ITS (Sigma-Aldrich), 20 ng/mL BMP2 (PeproTech), 30 ng/mL FGF4 (PeproTech), 20 ng/mL HGF (PeproTech), 10 ng/mL oncostatin M (PeproTech), and 10 μM dexamethasone (Sigma-Aldrich). Eight HLCs were investigated at each time point. Three independent experiments were performed for the cell analysis.

### Glucose-stimulated insulin secretion test

A glucose-stimulated insulin secretion test was performed according to previous reports^50–52^. Briefly, differentiated cells were stimulated by 2.2 or 22 mM glucose in Krebs-Ringer’s solution for 1 hour at 37 °C in a 5% CO_2_ incubator. Krebs-Ringer’s solution consisted of 129 mM NaCl, 1.2 mM MgSO_4_, 1.2 mM KH_2_PO_4_, 4.7 mM KCl, 5 mM NaHCO_3_, 2.5 mM CaCl_2_, 10 mM HEPES, and 1% bovine serum albumin (Sigma-Aldrich). After stimulation, the supernatant of each sample was collected, and human insulin concentrations were measured by a Human/Canine/Porcine Insulin Quantikine ELISA Kit (R&D Systems, Minneapolis, MN). The optical absorbance was measured by a SpectraMax i3 (Molecular Devices, Sun Jose, CA) and SoftMax Pro 7 (Molecular Devices). The stimulation index (SI) was calculated as the ratio of the insulin measurement after 22 mM glucose stimulation divided by the insulin measurement after 2.2 mM glucose stimulation. Three independent experiments were performed to evaluate GSIS (n = 3).

### Electron microscopy

Cells were prepared as reported previously^17^. Briefly, 3D-cultured IPCs were fixed in 0.1 M phosphate buffer with 2% paraformaldehyde and 2% glutaraldehyde at 4 °C overnight on day 21. After dehydration in graded ethanol solutions, the IPC samples were infiltrated by propylene oxide and resin (Nissin EM, Tokyo, Japan) for 1 hour. After transfer to fresh 100% resin, the IPC samples were polymerised at 60 °C for 48 hours and then sectioned at 70 nm thicknesses using an ultramicrotome (Leica, Vienna, Austria). The sections were stained with 2% uranyl acetate at room temperature for 15 minutes. After washing with distilled water, the samples were secondary stained with a Lead stain solution (Sigma-Aldrich) at room temperature for 3 minutes. Electron microscopy was performed at Tokai Electron Microscopy Inc. (Nagoya, Japan). Sections were observed under a JEM-1400 Plus transmission electron microscope (JEOL Ltd. Tokyo, Japan) at an acceleration voltage of 100 kV. Images were obtained by an EM-14830RUBY2 CCD camera (JEOL Ltd). As a control, we have included a human β-cell image obtained by electron microscopy (Fig. 1F, right, this image was reused in this study under an agreement with Elsevier^25^).

### Dithizone staining

Cultured cells (n = 8, each day of the evaluation) were stained by a dithizone solution. The dithizone solution consisted of 50 mg dithizone (Wako) per 5 mL dimethyl sulfoxide (Wako). The IPCs were incubated in the dithizone solution at 37 °C with 5% CO_2_ after washing with PBS three times. The stained samples were assessed by a BZ-X710 multi-purpose microscope (KEYENCE Engineering, Tokyo, Japan) and BZ-X Analyzer (KEYENCE Software, Japan).

### Zn^2+^ concentration assay

The culture supernatant was collected during medium changes. The Zn^2+^ concentration in each IPC sample was evaluated by a Metallo Assay kit zinc LS (Metallogenics Co., Chiba, Japan), according to manufacturer’s protocol. Briefly, 230 μl buffer, 12 μl sample, and 5 μl chelate colour solution were mixed and then transferred to a clear 96-well flat bottom plate (Corning Co.). Sample plates were analysed at 560 nm optical absorbance. The Zn^2+^ concentration was determined according to the optical absorbance of the standard sample (200 mg/dl) and blank sample (deionised distilled water). Optical absorbance were measured by a SpectraMax i3 (Molecular Devices) and SoftMax Pro 7 (Molecular Devices). Each Zn2+ concentration change was calculated as: ΔZn^2+^  = Zn^2+^ (supernatant) − Zn^2+^ (fresh culture medium). Eight IPCs were investigated at each time point. Three independent experiments were performed for this assay (n = 3).

### Immunohistochemical staining

IPC samples were fixed in formalin overnight and embedded in paraffin after embedding in iPGell (Genostaff, Tokyo, Japan). Four micrometre-thick sections of IPC samples and human kidney and pancreas (renal tubule cells and islets as negative and positive controls, respectively) were prepared. Usage of these resected specimens was approved by Tokushima University Hospital (Tokushima Clinical Trial Management System 2900-1, Dec 25, 2017). Specimen collection was performed in accordance with relevant guidelines/regulations. All patients had provided written informed consents. Sections were dewaxed, deparaffinised in xylene (Wako), and rehydrated through a series of graded alcohol solutions. Endogenous peroxidases were blocked with 0.3% hydrogen peroxidase (Wako) for 20 minutes. For antigen retrieval, the sections were boiled in EDTA buffer (pH 6.0) using a microwave for 15 minutes at 1400 W and then for 10 minutes at 700 W. The sections were incubated in Protein Serum Free (Dako, Burlington, Canada) for 10 minutes to prevent nonspecific antigen binding. Samples were incubated with primary antibodies for 1.5 hours at room temperature. Primary antibodies were: anti-insulin (aa287-299, LS-B129; 1:100, LSBio, Seattle, WA), anti-human ZnT8 (16169-1-AP, 1:50, Proteintech, Chicago, IL) and anti-human ZIP4 (20625-1-AP, 1:200, Proteintech). The sections were then incubated with a secondary antibody (EnVision Dual Link System-HRP, DAKO) for 1 hour at room temperature. Sections were developed in diaminobenzidine (Wako) and counterstained with Mayer’s haematoxylin (Muto pure chemicals, Tokyo, Japan). The stained sections were dehydrated in graded alcohol solutions and coverslipped.

### Immunofluorescence staining

Specimens of IPCs were sectioned, dewaxed, deparaffinised, and rehydrated. Blocking of endogenous peroxidases and retrieval of antigens were performed as described above. To prevent nonspecific antigen binding, the sections were incubated in 3% BSA for 1 hour. The sections were then incubated with an anti-human insulin antibody (#4590S, Cell Signaling Technology, Danvers, MA) as the primary antibody at 4 °C overnight. The sections were incubated with a secondary antibody, Alexa Fluor 555 goat anti-rabbit IgG (A-21244, 1:500, Invitrogen), after washing with PBS three times. Following washes with PBS, the sections were incubated with 4′,6-diamidino-2-phenylindole (28718-90-3, 1:2000, Santa Cruz Biotechnology, Paso Robles, CA) to detect nuclei. The stained samples were washed with PBS and coverslipped.

### Quantitative imaging analysis

Images of cells were digitally analysed by ImageJ (National Institute of Health, Bethesda, ML)^26^. In the analysis of dithizone staining, images of cells were converted to hue, saturation and value (HSV) models and filtered by hue thresholds of 7–20. The mean grey value of saturation of three random points in one spheroid area was measured in filtered images as the staining intensity^52^. In the analysis of immunohistochemical staining, after divided images into three channels, the images were filtered by hue thresholds of 20–28. The mean grey value of saturation of three random points in one spheroid area and background were measured in filtered images. The staining intensity of immunohistochemistry was calculated as the ratio of the mean grey value in the spheroid area divided by that of the background. Staining intensity data were calculated as the average of eight cell spheres (three random points of IPCs, n = 8).

### IPC transplantation into streptozotocin-induced diabetic nude mice

As an *in vivo* functional assay, transplantation of IPCs was undertaken as described in our previous report^17^. Briefly, 200 mg/kg body weight streptozotocin (STZ; Sigma-Aldrich) was dissolved in citrate buffer (pH 4.5) and administered to 5–6 week-old BALB/c nude mice (Charles River Japan, Yokohama, Japan) by intraperitoneal injection. STZ-induced DM nude mice were defined as those with blood glucose values over 350 mg/dl for two continuous readings or 400 mg/dl in one reading. Ninety-six IPCs were transplanted into the mesentery of STZ-induced DM mice, according to our previous method for IPC transplantation^17^. Sham mouse group mice (n = 4, administered normal saline intra-mesentery) and naïve nude mouse group (n = 4) were included. After transplantation, blood glucose values obtained using a Medisafe Fit kit (TERUMO, Tokyo, Japan) and body weight were recorded every 2 days, including sham and naïve nude mouse groups. All mice were bred in the animal facility at Tokushima University. Experiments and procedures were approved by the Animal Care and Use Committee of Tokushima University and performed in accordance with the NIH Guide for the Care and Use of Laboratory Animals.

### Quantitative reverse transcription-polymerase chain reaction analyses

Total RNA was extracted from cultured cells with an RNeasy Mini Kit (QIAGEN, Hilden, Germany), according to the manufacturer’s instructions. RNA purity was assessed using a Nano Drop ND-1000 spectrometer (Thermo Scientific). cDNA was synthesised from total RNA using a reverse transcription kit (QIAGEN). Quantitative reverse transcription-polymerase chain reaction analysis was performed using TaqMan Gene Expression Assays in 7500 real-time polymerase chain reaction system with StepOne Plus software (Applied Biosystems, Foster City, CA). The gene expression level was normalised to the GAPDH gene expression level. Primers for SOX17 (Hs00751752_s1, Applied Biosystems) and NGN3 (Hs01875204_s1, Applied Biosystems) were used in this study.

### Statistical analysis

Statistical calculations were performed using SPSS statistics version 24 (IBM, Chicago, IL) or State Mate III (ATMS Co., LTD. Tokyo, Japan). Sample data were compared by the Mann-Whitney U-test, Student’s and Welch’s t-tests, or chi-squared test. Comparisons between multiple groups were performed by one-way ANOVA, Bonferroni’s test, and Fisher’s least significant difference. P-values of less than 0.05 were considered as significant.

## Data Availability

The datasets used and/or analysed in this study are available from the corresponding author upon reasonable request.
